# Trajectories and predictors of women’s health-related quality of life during pregnancy: A large longitudinal cohort study

**DOI:** 10.1371/journal.pone.0194999

**Published:** 2018-04-03

**Authors:** Guannan Bai, Hein Raat, Vincent W. V. Jaddoe, Eva Mautner, Ida J. Korfage

**Affiliations:** 1 Department of Public Health, Erasmus MC-University Medical Center Rotterdam, Rotterdam, South Holland, the Netherlands; 2 The Generation R Group, Erasmus MC- University Medical Center Rotterdam, Rotterdam, South Holland, the Netherlands; 3 Department of Epidemiology, Erasmus MC-University Medical Center Rotterdam, Rotterdam, South Holland, the Netherlands; 4 Department of Paediatrics, Erasmus MC- University Medical Center Rotterdam, Rotterdam, South Holland, the Netherlands; 5 Department of Obstetrics and Gynaecology, Medical University of Graz, Auenbruggerplatz 14, Graz, Austria; Iranian Institute for Health Sciences Research, ISLAMIC REPUBLIC OF IRAN

## Abstract

The objective of this study was to identify distinct trajectories and their predictors of health-related quality of life (HRQOL) of women during pregnancy in a prospective mother and child cohort. Analyses were based on 3936 Dutch pregnant women in Rotterdam area, the Netherlands. Information on potential predictors was collected in early pregnancy by questionnaire. Latent Class Mixture Modelling and Multinomial Logistic Regression were applied to assess the trajectory and predictors of HRQOL during pregnancy. HRQOL was measured by SF-12 in early, mid- and late pregnancy; physical and mental component summary (PCS-12/MCS-12) scores were calculated. Four physical HRQOL trajectories were identified: a healthy trajectory (‘healthy’) in 63.3%, consistently low (‘vulnerable’) in 10.8%; a small increase (‘recovering’) in 12.8% and a large decrease (‘at risk’) in 13.1%. Three mental HRQOL trajectories were identified: a healthy trajectory (‘healthy’) in 86.1%; a large increase (‘recovering’) in 7.5%; and a large decrease (‘at risk’) in 6.4%. Compared with healthy trajectories, the likelihood of following the ‘vulnerable’ physical HRQOL trajectory rather than a healthy trajectory was increased by daily fatigue(OR: 4.82[2.76, 8.40]), pelvic pain (OR:4.76[2.91, 7.78]) and back pain (OR:5.29[3.21, 8.70]); pregnancy-specific anxiety increased the likelihood of following the ‘at risk’ mental HRQOL trajectory (OR:7.95[4.84, 13.05]). Healthy physical and mental HRQOL trajectories during pregnancy were most common. Predictors indicative of poor HRQOL trajectories included pregnancy-related symptoms and anxiety.

## Introduction

Health-related quality of life (HRQOL) is a multidimensional term referring to the health aspects of quality of life, encompassing physical and occupational functions, psychological state, social interaction and somatic sensation.[1] Women’s HRQOL is acknowledged as a critical concept in the childbearing period.[2, 3] It provides a broad view of women’s experience during pregnancy.

Many studies have demonstrated associated factors of HRQOL in pregnancy. For instance, young maternal age, low education, financial dissatisfaction, unplanned pregnancy, pregnancy-related symptoms, depression and domestic violence may be associated with low HRQOL;[4–9] while participation in physical activities and social support may be associated with high HRQOL.[10, 11] However, most study designs are cross-sectional, providing limited insights into HRQOL trajectories during pregnancy.

Two studies have reported changes of HRQOL during pregnancy.[12, 13] Haas et al. reported a decrease of physical functioning during pregnancy but did not conduct longitudinal analysis to identify predictors of the trend.[12] Chang et al. found that physical functioning was poorest in late pregnancy whereas mental health was poorest in early pregnancy; longitudinal analysis demonstrated that stage of pregnancy, parity, previous infertility, assisted reproduction, unplanned pregnancy and medical conditions were predictors of HRQOL during pregnancy.[13] Other longitudinal studies relevant to women’s HRQOL in perinatal period only measured HRQOL in late pregnancy and then after delivery.[3, 14–16]

A population may include different subgroups of individuals sharing a common, underlying pattern of HRQOL change over time (latent class). There is very limited data on the distinct trajectories of HRQOL during pregnancy. Identifying the potential distinct trajectories of HRQOL during pregnancy and their predictors may be of benefit to health professionals and pregnant women, as well as to policy makers, so that women more likely to have greater need of healthcare services can be identified and interventions can be targeted at more specific risk factors for the poor HRQOL trajectory. To help reduce this knowledge gap, we conducted the present study by analysing data from a large, population-based prospective mother and child cohort in the Netherlands, aiming to identify distinct trajectories of HRQOL from early to late pregnancy and to assess predictors of poor HRQOL trajectories in the early phase of pregnancy. We used a latent class approach, assuming that a population of pregnant women may include different subgroups of individuals sharing a common, underlying pattern of HRQOL change over time.

## Methods

### Data source

Data were obtained from the Generation R Study, a prospective population-based mother and child cohort from fetal life until adulthood. The Generation R Study has been described previously in detail.[17–20] Briefly, the cohort includes 9778 (response rate 61%) mothers with a delivery date from April 2002 until January 2006 and their children, living in the Rotterdam area, the Netherlands. [19] Although when Generation R was being set up the aim was to enrol women in early pregnancy (gestational age < 18 weeks), enrolment was possible until parturition. 7069 mothers were enrolled in early pregnancy, 1594 mothers in mid-pregnancy (gestational age 18–25 weeks), 216 mothers in late pregnancy (gestational age ≥25 weeks) and 899 mothers at parturition. Physical examinations and four postal questionnaires were planned in early, mid- and late pregnancy. The study was conducted in accordance with the World Medical Association’s Helsinki guidelines and was approved by the Medical Ethical Committee of the Erasmus Medical Center, University Medical Center Rotterdam.[21] Written consent had been obtained from all of the participating women.[21]

### Study population

Of the 8879 mothers enrolled in prenatal phase, we excluded pregnancies with the following outcomes: twin birth (n = 97), induced abortion (n = 29), fetal deaths before 20 weeks of gestation (n = 75), loss to follow-up pregnancy outcomes (n = 45). Additionally, we excluded mothers who were not Dutch (n = 4163) and mothers for whom data on ethnic background was missing (n = 473). Finally, we excluded mothers with missing data for three measurements of SF-12 (n = 61). This left 3936 mothers with at least one measurement of SF-12 in early, mid- and/or late pregnancy, who were eligible for analysis in the present study (see S1 Fig).

### Health-related quality of life

HRQOL was measured using the SF-12 questionnaire at three waves: early, mid- and late pregnancy. SF-12 includes 12 items regarding eight scales: physical functioning, role limitations due to physical problems, bodily pain, general health, vitality, social functioning, role limitation due to emotional problems and perceived mental health. SF-12 is a reliable and well-validated instrument to measure HRQOL and is widely used in studies with large sample sizes.[22] Some items were recoded and the raw score of each scale was transformed into 0 (the worst) to 100 (the best) before we calculated the raw Physical Component Summary (PCS-12) score and the raw Mental Component Summary (MCS-12) score. Finally the raw PCS-12and MCS-12 scores were transformed into the standard scores based on the normalised algorithms from the United States general population with the mean value of 50 and the standard deviation of 10.[23]

### Potential predictors

We measured 18 variables in early pregnancy as potential predictors of women’s HRQOL trajectory during pregnancy, including maternal/gestational age, education, marital status, household income, parity, planned pregnancy, body mass index (BMI), maternal smoking and drinking, pregnancy-related physical symptoms (i.e. headache, fatigue, sleeping badly, pelvic pain, back pain, nausea, vomiting) and pregnancy-specific anxiety.

Information on all variables was collected by the questionnaire at intake. Education was categorised into four successive levels based on the Dutch Standard Classification of Education: high (Master’s degree or PhD), mid-high (higher vocational training, Bachelor’s degree), mid-low (>3 years general secondary school, intermediate vocational training) and low (no education, primary school, lower vocational training, intermediate general school, or 3 years or less general secondary school).[24] Household income was coded as low (< 2200 euros per month) and high (≥2200 euros per month). BMI was based on women’s height and weight measured at intake. Maternal smoking and alcohol use were measured with three options ‘non-smokers/teetotal’, ‘stopped when pregnancy was known’ and ‘continued to smoke/drink during pregnancy’. The frequency of pregnancy-related physical symptoms (i.e. fatigue, pelvic pain, back pain, sleeping badly, nausea, vomiting, headache) was measured in early pregnancy on a five-point Likert scale: ‘daily’, ‘a few days a week’, ‘once per week’, ‘less than once per week’ or ‘never’. In the multinomial logistic regression models, we lumped the frequency of symptoms into three or two categories to avoid extremely small subgroups. Pregnancy-specific anxiety was assessed by an adapted version of the Pregnancy Outcome Questionnaire in early pregnancy.[25] This version consisted of 13 items that were rated on four-point scales ranging from ‘0’ (almost never) to ‘3’ (almost always). Total scores were calculated by summing the item scores and dividing by the number of endorsed items.[26] In the present study, the internal consistency was α = 0.67.

### Statistical analyses

We applied Latent Class Mixture Modelling (LCMM) to assess the distinct trajectories of women’s HRQOL during pregnancy.[27, 28] First, a preliminary LCMM analysis was conducted in R Studio (R x64 3.3.2) without covariates, to identify the optimal number of latent classes (distinct trajectories) for PCS-12 scores and MCS-12 scores. A distinct trajectory consists of a group of individuals who share a common underlying pattern of HRQOL change over time.[29] First we tried one latent class, then two latent classes, and so on. The optimal number of latent classes was evaluated by model fit statistics, i.e. the Akaike information criterion (AIC) and Bayes information criterion (BIC). Lower values indicate a better-fitting model. The optimal number of latent classes is achieved if adding one latent class fails to produce a better model fit.[27]

Next, we performed a descriptive analysis of the characteristics of the study population. The chi square test for categorical variables and one-way ANOVA for continuous variables were applied to describe differences in covariates across latent classes.

Finally, all significant predictors identified in the second step were incorporated into the final model, using multinomial logistic regression. We have only included the cases with complete data on these predictor variables for regression analyses (n = 2852 and n = 2803, respectively). The optimal latent classes of PCS-12 and MCS-12, identified in the first step, were regarded as outcome variables. To explore the potential bias that may result from only including women with complete data on predictor variables, we assessed differences of characteristics between women who were included in the regression analyses and women who were excluded from the regression analyses using two independent t-tests and Chi Square tests. Additionally, we evaluated whether the HRQOL trajectories differed between the women included in the regression analyses and those excluded from the analyses using Chi Square tests.

All the analyses were conducted in SPSS 21.0 (IBM Corp., Armonk, NY, USA). Significance was indicated at p <0.05.

## Results

The mean age of women at intake was 31 years; mean gestational age at intake was around 14 weeks. 59.8% women were in their first pregnancy; 18.7% reported unplanned pregnancy. S1 Table presents the general characteristics of the study population.

### Determining the latent classes

**Fig 1:**
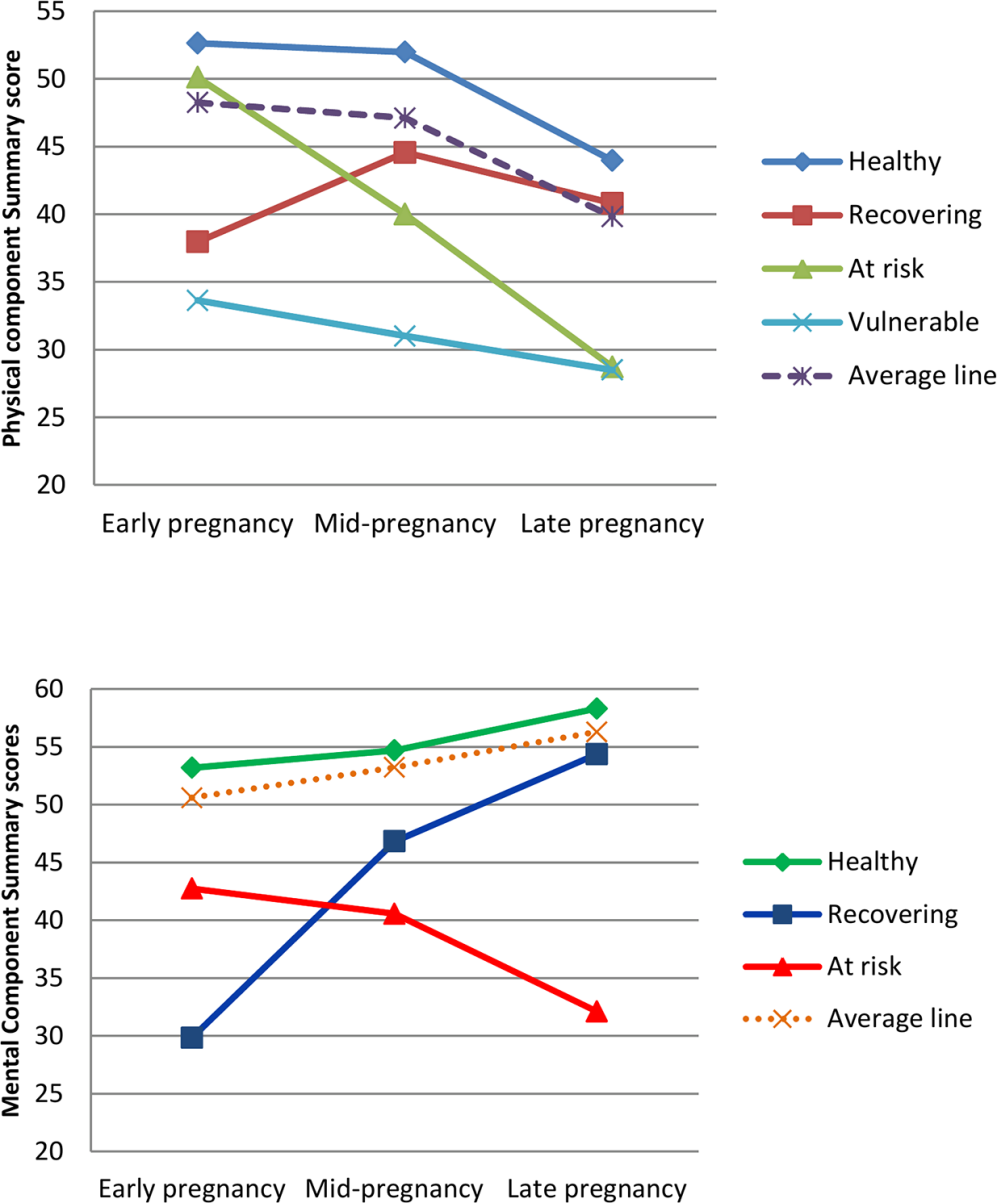
Trajectories of Physical/Mental Component Summary scores during pregnancy.

As indicated by the model fit indices (see Table 1), four latent classes (distinct trajectories) of PCS-12 and three latent classes of MCS-12 were identified as the optimal numbers of latent classes by LCMM. S2 Table presents the means of PCS-12 and MCS-12 scores across the latent classes. Fig 1 illustrates these distinct trajectories. Regarding PCS-12, the first trajectory contained more than half of the women (n = 2491, 63.3%) and represented a healthy trajectory of physical HRQOL during pregnancy (termed ‘healthy’); the second trajectory, termed ‘recovering’, contained 505 women (12.8%) and represented an increase in physical HRQOL during pregnancy; the third trajectory (n = 516, 13.1%), termed ‘at risk’, was characterised by a significant decline in physical HRQOL; the fourth trajectory (n = 424, 10.8%), termed ‘vulnerable’, was characterised by consistently low mean scores of PCS-12 during pregnancy. Regarding MCS-12, the first trajectory contained the majority of women (n = 3388, 86.1%), representing a consistent and slight increase in means during pregnancy (termed ‘healthy’); the second trajectory (n = 295, 7.5%), termed ‘recovering’, was characterised by a significant increase in mean scores over time; the third trajectory (n = 253, 6.4%), termed ‘at risk’, was characterised by a significant decrease in mean scores over time.

**Table 1:** Fit indices used to identify number of latent classes.

	Physical Component Summary		Mental Component Summary	
Number of latent class	AIC	BIC	AIC	BIC
**1**	71538.75	71570.14	70648.4	70679.79
**2**	71055.89	71112.39	69589.75	69646.25
**3**	70972.31	71053.92	68467.27	68548.88
**4**	70710.32	70817.05	68475.27	68581.99
**5**	70718.32	70850.16		

### Predictors of the trajectory of HRQOL during pregnancy

S3 and S4 Tables show the distribution of covariates across latent classes of PCS-12 and MCS-12 during pregnancy. Significant covariates were included in the multinomial logistic regression models by using the healthy trajectories of PCS-12 and MCS-12 as the reference. Tables 2 and 3 present Odds Ratios (ORs) for all the predictors of PCS-12 and MCS-12, respectively.

**Table 2:** Significant predictors of trajectories of Physical Component Summary scores during pregnancy (n = 2852).

Predictors	OR (95% CI)		
	Vulnerable	At risk	Recovering
**Gestational age at intake**	1.07 (1,03, 1,10)**	1.00 (0.97, 1.04)	1.03 (0.99, 1.06)
**Maternal educational level**			
**High**	reference	reference	reference
**Mid-high**	1.02 (0.70, 1.49)	1.34 (0.99, 1.82)	1.11 (0.83, 1.48)
**Mid-low**	1.29 (0.89, 1.87)	1.28 (0.93, 1.75)	0.94 (0.68, 1.28)
**low**	0.80 (0.50, 1.29)	0.69 (0.45, 1.06)	0.64 (0.41, 1.00)
**Parity**			
**multiparity**	Reference	reference	reference
**Null parity**	0.87 (0.65, 1.16)	0.71 (0.56, 0,90)**	1.35 (1.05, 1.74)*
**BMI at intake**	1.06 (1.03, 1.10)**	1.06 (1.03, 1,10)**	1.01 (0.98, 1.04)
**Maternal smoking in early pregnancy**			
**Non-smoker**	reference	reference	reference
**Smoked until pregnancy confirmed**	0.82 (0.52, 1.29)	1.38 (0.98, 1.93)	0.86 (0.57, 1.31)
**Continued smoking during pregnancy**	0.45 (0.27, 0,74)**	1.25 (0.86, 1.82)	0.42 (0.24, 0.69)**
**Chronic conditions in previous year**			
**None**	reference	reference	reference
**One**	1.33 (0.98, 1.80)	1.36 (1.06, 1.76)*	0.94 (0.73, 1.22)
**≥Two**	1.64 (1.09, 2.48)*	1.89 (1.34, 2.69)**	1.20 (0.83, 1.74)
**Headache in early pregnancy**			
**≤Once a week**	reference	reference	reference
**Daily/few days a week**	2.64 (1.83, 3.80)**	1.33 (0.91, 1.94)	1.64 (1.13, 2.36)**
**Fatigue in early pregnancy**			
**≤ Once a week**	reference	reference	reference
**A few days a week**	1.82 (1.03, 3.21)*	1.55 (1.06, 2.28)*	2.20 (1.40, 3.45)**
**Daily**	4.82 (2.76, 8.40)**	2.61 (1.77, 3.85)**	3.71 (2.36, 5.84)**
**Pelvic pain in early pregnancy**			
**≤Once a week**	reference	reference	reference
**Daily/ a few days a week**	4.76 (2.91, 7.78)**	2.86 (1.74, 4.71)**	1.82 (1.02, 3.22)*
**Back pain in early pregnancy**			
**≤Once a week**	reference	reference	reference
**A few days a week**	2.04 (1.40, 2.95)**	1.98 (1.44, 2.72)**	1.61 (1.14, 2.26)**
**Daily**	5.29 (3.21, 8.70)**	1.52 (0.85, 2.73)	3.11 (1.82, 5.30)**
**Nausea in early pregnancy**			
**≤Once a week**	reference	reference	reference
**A few days a week**	1.13 (0.79, 1.63)	1.07 (0.85, 1,41)	1.98 (1.46, 2.68)**
**Daily**	2.26 (1.62, 3.18)**	1.44 (1.08, 1.93)*	3.33 (2.46, 4.51)**
**Pregnancy-specific anxiety**	2.10 (1.34, 3.29)**	1.27 (0.85, 1.87)	1.64 (1.10, 2.43)*

Values are presented as ORs using the healthy trajectory as a reference category.

*p<0.05

**p<0.01

**Table 3:** Significant predictors of trajectories of Mental Component Summary scores during pregnancy (n = 2803).

Predictors	OR (95% CI)	
	At risk	Recovering
**Maternal age at intake**	1.06 (1.02, 1.10)**	1.06(1.02, 1.09)**
**Monthly household income (€)**		
**>2200**	reference	reference
**≤2200**	2.06 (1.45, 2.94)**	1.39 (0.99, 1.94)
**Planned pregnancy**		
**Yes**	reference	reference
**No**	2.60 (1.80, 3.74)**	1.39 (0.96, 2.02)
**Maternal smoking during pregnancy**		
**Non-smoker**	reference	reference
**Smoked until pregnancy confirmed**	1.40 (0.86, 2.24)	2.18 (1.50, 3.18)**
**Continued to smoke during pregnancy**	2.08 (1.37, 3.18)**	1.32 (0.82, 2.11)
**Nausea in early pregnancy**		
**≤Once a week**	reference	reference
**A few days a week**	1.32 (0.89, 1.96)	1.62 (1.12, 2.32)*
**Daily**	1.67(1.13, 2.46)*	2.10 (1.48, 2.99)**
**Sleeping badly in early pregnancy**		
**≤Once a week**	reference	reference
**A few days a week**	1.88 (1.32, 2.68)**	1.27 (0.91, 1.77)
**Daily**	2.52 (1.51, 4.21)**	2.06 (1.26, 3.37)**
**Pregnancy-specific anxiety**	7.95 (4.84, 13.05)**	5.33 (3.36, 8.43)**

Values are presented as ORs using the healthy trajectory as a reference category.

*p<0.05

**p<0.01

### Physical HRQOL trajectories

#### Vulnerable trajectory vs. healthy trajectory

Women who were enrolled in the study at later gestational stage and who had higher body weights or higher levels of pregnancy-specific anxiety were more likely to follow the ‘vulnerable’ trajectory than those who were enrolled earlier, had lower body weight, or lower levels of anxiety. Those with more than two chronic conditions or with pregnancy-related physical symptoms (i.e. headache, fatigue, pelvic pain, back pain and nausea) also had higher odds of following a ‘vulnerable’ trajectory. Dose effects were observed for chronic condition, fatigue, back pain and nausea. Women who continued to smoke even though they were aware of their pregnancy were less likely to follow the ‘vulnerable’ trajectory (OR:0.45, 95% CI: 0.27, 0.74).

#### At risk trajectory vs. healthy trajectory

The odds of following the ‘at risk’ trajectory of physical HRQOL were significantly higher in women with one or more chronic conditions, fatigue, pelvic pain, back pain and nausea than in women without these conditions or symptoms. Higher BMI also increased this likelihood (OR:1.06, 95%CI:1.03, 1.10). However, being pregnant for the first time decreased this likelihood (OR: 0.71, 95%CI: 0.56, 0.90).

#### Recovering trajectory vs. healthy trajectory

Women who continued to smoke even though they were aware of the pregnancy were less likely to follow the ‘recovering’ trajectory (OR:0.42, 95% CI: 0.24, 0.69). Women who were in their first pregnancy, or had pregnancy-related physical symptoms (i.e. headache, fatigue, pelvic pain, back pain and nausea) and pregnancy-specific anxiety were more likely to follow a ‘recovery’ trajectory.

### Mental HRQOL trajectories

#### At risk trajectory vs. healthy trajectory

Women who were older and had higher anxiety levels were more likely to follow the ‘at risk’ trajectory than the ‘healthy’ trajectory. The most notable finding was that a one-point change in the pregnancy-specific anxiety measure resulted in a 7.95-fold increase (OR: 7.95, 95% CI: 4.84, 13.05) in the odds of classification into the ‘at risk’ trajectory. Women who had a low household income, unplanned pregnancy, nausea, were sleeping badly or continued to smoke even though they were aware of their pregnancy were also more likely to follow the ‘at risk’ trajectory.

#### Recovering vs. healthy

The odds of falling into the ‘recovering’ trajectory were significantly higher among women who were older, stopped smoking when the pregnancy was known, presented with nausea and sleeping badly, and had higher anxiety levels. When women stopped smoking because of the awareness of pregnancy, the odds of following the recovering trajectory increased significantly (OR: 2.18, 95% CI: 1.50, 3.18).

S5 and S6 Tables show that the excluded women were younger, more often single, more often with lower educational level, lower household income, higher BMI, and they more often reported smoking during pregnancy, having chronic condition(s), having pregnancy-related physical symptoms (such as headache, fatigue, nausea, vomiting, sleeping difficulty, pelvic pain and back pain) and reported a higher pregnancy-specific anxiety (p<0.05). Additionally, S7 and S8 Tables demonstrated that there were no significant differences with regard to the distribution of both physical and mental HRQOL trajectories between the women included in the analyses and those excluded from the analyses (p>0.05).

## Discussion

This study identified distinct trajectories of physical and mental HRQOL during pregnancy in a large community sample of pregnant Dutch women. More than 60% of the women had a healthy physical HRQOL level, and the majority of women (86%) had healthy levels of mental HRQOL during the entire pregnancy, which is a positive finding. However, by comparison with women following the healthy trajectory, women with poor HRQOL trajectories were found to have different patterns of characteristics. Therefore, assisting them to modify the factors leading to worse HRQOL may prevent the deterioration of HRQOL in pregnancy.

### Trajectories of physical HRQOL

Nausea and fatigue are the most common somatic symptoms in early pregnancy and they may be associated with lower physical HRQOL in early pregnancy.[5, 30] So far, little is known about the long-term impact of fatigue and nausea on physical HRQOL during pregnancy. Our study showed that daily presence of fatigue and nausea in early pregnancy may be associated with experiencing a suboptimal physical HRQOL during pregnancy. Even though pelvic/back pain is not as common in early pregnancy as nausea and fatigue, their impact on physical HRQOL trajectory is significant. Therefore, management of these pregnancy-related physical symptoms from early pregnancy is warranted and may prevent physical HRQOL decreasing over time in pregnancy.

Additionally, our study indicated that higher BMI may be associated with a decrease of physical HRQOL during pregnancy. A longitudinal study in Finland yielded a similar finding: the decrease of HRQOL during pregnancy was significantly larger in the obese group.[14] Not being pregnant for the first time and presence of chronic conditions increased the likelihood of following the ‘at risk’ trajectory. It has been suggested that women with higher parity status may have lower physical HRQOL.[31] So far, little is known about the impact of chronic conditions during pregnancy on HRQOL. The existing studies focus on specific conditions, such as gestational diabetes, showing that pregnant women with chronic conditions may have worse HRQOL in both the short and long term.[32] Chronic conditions in pregnancy, such as high blood pressure, diabetes and heart disease may put women at higher risk of pregnancy complications.[33] Our findings suggest that pregnancy-specific anxiety may have impacted on how women perceive their physical quality of life during pregnancy. Women with high levels of trait anxiety may be hypervigilant during pregnancy and inclined to interpret ambiguous stimuli such as inconclusive test results or bodily sensations like cramp as threatening.[34]

Unexpectedly, we found that women who continued to smoke when they were aware of the pregnancy were less likely to follow a trajectory of suboptimal physical HRQOL during pregnancy. We cannot explain this finding. We stress that in our study, physical HRQOL refers to the perceived physical quality of life rather than measured physical health. There is no doubt that smoking negatively affects mother’s physical health and also fetal health.[35] We recommend further research on the association between maternal smoking and HRQOL.

### Trajectories of mental HRQOL

Our study showed that various factors may predict the decrease of mental HRQOL during pregnancy, such as low household income, unplanned pregnancy, continuation with smoking and presence of nausea, sleeping badly and pregnancy-specific anxiety. Nilna et al. reported that women in early pregnancy who were financially insecure tended to have lower HRQOL than women who were financially secure, and this may influence the later health or wellbeing of mothers.[4] Unplanned pregnancy has been found to be a significant risk factor for women’s mental health.[36, 37] Furthermore, the suggestion that unplanned pregnancy may affect women’s mental health more than their physical health [37] is supported by our results. Our finding that nausea and sleeping badly were also associated with the decreasing of mental HRQOL is consistent with findings of previous studies.[5, 6, 30, 38] Disrupted sleep is related to peripartum mood disorders and these are associated with a significant reduction in HRQOL.[7] The most notable factor affecting mental HRQOL in our study was pregnancy-specific anxiety. It can be thought of as the interaction between a woman’s general predisposition to anxious emotional states and the conditions of her pregnancy, including medically risky conditions and psychosocial factors.[34] Pregnancy-specific anxiety is related to previous negative pregnancy experience and may be associated with other psychosocial variables such as depressive symptoms, stress and low self-esteem.[39] Guardino et al. have suggested that regardless of its origin, anxiety during pregnancy poses a greater risk than medical conditions and traditional risk factors.[34]

Women who stopped smoking when they were aware of the pregnancy were also more likely to have an improving mental HRQOL during pregnancy; and women who continued smoking even though they were aware of their pregnancy were more likely to have a decreasing mental HRQOL during pregnancy. This finding is consistent with previous studies on maternal smoking during pregnancy and women’s mental health: women who smoked during pregnancy were more likely to have worse mental health and to have received treatment for mental disorders.[40, 41]

The present study has identified various patterns of predictors for physical and mental HRQOL trajectories during pregnancy which health professionals could take into account when developing targeted interventions. Two aspects in particular that should be targeted in health promotion strategies are management of pregnancy-related physical symptoms and alleviating pregnancy-specific anxiety.

### Strengths and limitations

To our knowledge, this is the first study to apply LCMM to the study of HRQOL trajectories during pregnancy in a large community sample. Usually, the entire population is analysed and the average trajectory identified, which is likely to be similar to the trajectory of the majority. However, in a heterogeneous and diverse population, different trajectories may exist. LCMM enables the identification of the distinct underlying trajectories. A second strength is that the present study is a prospective study in a large population-based community sample of 3936 women, and information was available on a comprehensive set of covariates. This enabled the identification of clearly distinct trajectories and of predictors for each trajectory.

Several limitations should be taken into account. As is to be expected in a prospective cohort study, there are several bias should be considered. The overall response rate in the entire Generation R Study was 61%.[19] Differences between women who accepted the invitation to participate and those who did not may lead to non-response bias. In general, the women participating in the Generation R Study are relatively healthier than the women in the source population.[20] Moreover, to assess the predictors of suboptimal HRQOL trajectories, we excluded study participants with missing values on the potential predicting variables from regression analyses. Compared with the included women, the excluded women were younger, more often single, more often with lower educational level, lower household income, higher BMI, and they more often reported smoking during pregnancy, having chronic condition(s), having pregnancy-related physical symptoms and reported a higher level of pregnancy-specific anxiety. Therefore, our results should be interpreted with caution. There were no significant differences regarding physical and mental HRQOL trajectories between the included women and the excluded women. In the present study, we only included women with a Dutch ethnic background in the analyses since we aimed for a more homogenous population to assess the trajectories of HRQOL for the first time. Therefore, the results in non-Dutch populations are unknown. Now that we are able to identify trajectories, we recommend repeating this study in large study populations with heterogeneous backgrounds to confirm or reject our findings.

## Conclusion

Physical and mental HRQOL trajectories during pregnancy differ, with the most common being healthy trajectories. The predictors we identified as being indicative of poor HRQOL trajectories included pregnancy-related symptoms and anxiety. Clinicians and other health professionals should recognise the predictors of adverse HRQOL trajectories during pregnancy, and collaborate across disciplines to address them in an early stage to prevent disparities in HRQOL becoming established.

## Supporting information

S1 STROBE Checklist STROBE statement. (DOC)

S1 Fig Flow chart of population for analysis in this study. (DOCX)

S1 Table General characteristics of the study population (n = 3936). (DOCX)

S2 Table Mean scores of Physical/Mental Component Summary scales across latent classes during pregnancy. (DOCX)

S3 Table Distribution of covariates across latent classes of Physical Component Summary scores during pregnancy (n = 3936). (DOCX)

S4 Table Distribution of covariates across latent classes of Mental Component Summary scores during pregnancy (n = 3936). (DOCX)

S5 Table Differences in characteristics between women included in analyses for identifying predictors for physical HRQOL trajectories (n = 2852) and women excluded analyses (n = 1084). (DOCX)

S6 Table Differences in characteristics between women included in analyses for identifying predictors for mental HRQOL trajectories (n = 2803) and women excluded analyses (n = 1133). (DOCX)

S7 Table Distribution of physical HRQOL trajectories between women included in analyses for identifying predictors for physical HRQOL trajectories (n = 2852) and women excluded from analyses (n = 1084). (DOCX)

S8 Table Distribution of mental HRQOL trajectories between women included in analyses for identifying predictors for mental HRQOL trajectories (n = 2803) and women excluded from analyses (n = 1133). (DOCX)
