# Supplementary material for: Trajectories and predictors of women’s health-related quality of life during pregnancy: A large longitudinal cohort study
Source: PLoS One. 2018 Apr 3;13(4):e0194999. doi: 10.1371/journal.pone.0194999 (PMC5882096; doi:10.1371/journal.pone.0194999)
Supplement: S1 Fig — (DOCX) [file pone.0194999.s002.docx]

Mothers enrolled in the entire Generation R Cohort

N=9778

Excluded: mothers enrolled at parturition (n=899)

Mothers enrolled in prenatal period

N=8879

Excluded: mothers with twin birth (n=97), induced abortion (n=29), fetal death before 20 weeks of gestation (n=75), loss to follow-up pregnancy outcomes (n=45)

Mothers with singleton live birth

N=8633

Excluded: mothers who were not Dutch (n=4163) and women with missing information on the ethnic background (n=473)

Mothers with Dutch ethnicity

N=3997

Excluded: mothers with missing information on three measurements of SF12 (n=61)

Mothers eligible for the present study:

Women with at least one measurement of SF12 in early, mid- and/or late pregnancy: N=3936

**S1 Fig.**
