# Supplementary material for: Trajectories and predictors of women’s health-related quality of life during pregnancy: A large longitudinal cohort study
Source: PLoS One. 2018 Apr 3;13(4):e0194999. doi: 10.1371/journal.pone.0194999 (PMC5882096; doi:10.1371/journal.pone.0194999)
Supplement: S1 Table — (DOCX) [file pone.0194999.s003.docx]

S1 Table

| **Characteristics** | **values** |
| --- | --- |
| **Maternal age at intake** | 31.29±4.50 |
| **Gestational age at intake** | 14.50±3.72 |
| **Maternal educational level** |  |
| **High** | 1285 (32.6) |
| **Mid-high** | 962 (24.4) |
| **Mid-low** | 1004 (25.5) |
| **Low** | 637 (16.2) |
| ***missing*** | *48* |
| **Marital status** |  |
| **Married/cohabiting** | 3542 (92.5) |
| **Single** | 287 (7.5) |
| ***missing*** | *107* |
| **Parity** |  |
| **Nullpara** | 2344 (59.8) |
| **Multipara** | 1581 (40.3) |
| ***missing*** | *11* |
| **Monthly household income (€)** |  |
| **≤2200** | 927 (26.6) |
| **>2200** | 2563 (73.4) |
| ***missing*** | *446* |
| **Planned pregnancy** |  |
| **No** | 690 (18.7) |
| **Yes** | 3002 (81.3) |
| ***missing*** | *244* |
| **BMI at intake** | 24.33±4.14 |
| ***missing*** | *18* |
| **Maternal smoking in early pregnancy** |  |
| **Non-smoker** | 2666 (74.6) |
| **Smoked until pregnancy confirmed** | 452 (12.5) |
| **Continued smoking in pregnancy** | 467 (12.9) |
| ***Missing*** | *318* |
| **Maternal drinking in early pregnancy** |  |
| **Teetotal during pregnancy** | 1496 (41.2) |
| **Drank until pregnancy confirmed** | 1262 (32.1) |
| **Continued drinking in pregnancy** | 870 (24.0) |
| ***Missing*** | *308* |
| **Chronic conditions in the previous year** |  |
| **None** | 1950 (55.7) |
| **One** | 1102 (31.5) |
| **≥ Two** | 449 (12.8) |
| ***missing*** | *435* |
| **Headache** |  |
| **Daily/ Few days a week** | 428 (12.0) |
| **≤ Once a week** | 3140 (88.0) |
| ***missing*** | *368* |
| **Fatigue** |  |
| **Daily** | 1517 (42.1) |
| **Few days a week** | 1497 (41.6) |
| **≤ Once a week** | 588 (16.3) |
| ***missing*** | *334* |
| **Sleeping badly** |  |
| **Daily** | 254 (7.1) |
| **Few days a week** | 861 (24.1) |
| **≤ Once a week** | 2457 (68.8) |
| ***missing*** | *364* |
| **Pelvic pain** |  |
| **Daily/ Few days a week** | 209 (6.1) |
| **≤ Once a week** | 3379 (94.2) |
| ***missing*** | *348* |
| **Back pain** |  |
| **Daily** | 221 (6.1) |
| **Few days a week** | 525 (14.6) |
| **≤ Once a week** | 2853 (79.3) |
| ***missing*** | *337* |
| **Nausea** |  |
| **Daily** | 1008 (28.0) |
| **Few days a week** | 1022 (28.3) |
| **≤ Once a week** | 1576 (43.7) |
| ***Missing*** | *330* |
| **Vomiting** |  |
| **Daily** | 178 (5.0) |
| **Few days a week** | 335 (9.3) |
| **≤ Once a week** | 3076 (85.7) |
| ***missing*** | *347* |
| **Pregnancy-specific anxiety** | 0.76±0.32 |
| ***missing*** | *371* |

*Values in this table are means, standard deviations, numbers and percentages.
