# Supplementary material for: Trajectories and predictors of women’s health-related quality of life during pregnancy: A large longitudinal cohort study
Source: PLoS One. 2018 Apr 3;13(4):e0194999. doi: 10.1371/journal.pone.0194999 (PMC5882096; doi:10.1371/journal.pone.0194999)
Supplement: S2 Table — (DOCX) [file pone.0194999.s004.docx]

S2 Table

|  | **early pregnancy (n=3391)** | **mid-pregnancy (n=3429)** | **late pregnancy (n=3368)** | **P value (early vs. mid-)** | **P value (mid- vs. late)** |
| --- | --- | --- | --- | --- | --- |
| **PCS** |  |  |  |  |  |
| **Healthy** | 52.64 (5.09) | 51.99 (5.27) | 43.97 (5.80) | <0.001 | <0.001 |
| ***Number (%)*** | *2127 (62.7)* | *2149 (62.7)* | *2118 (62.9)* |  |  |
| **Recovering** | 37.96 (3.87) | 44.56 (6.32) | 40.81 (6.21) | <0.001 | <0.001 |
| ***Number(%)*** | 478 (14.1) | 439 (12.8) | 426 (12.6) |  |  |
| **At risk** | 50.12 (5.18) | 40.01 (6.88) | 28.72 (5.81) | <0.001 | <0.001 |
| ***Number(%)*** | 430 (12.7) | 462 (13.5) | 475 (14.1) |  |  |
| **Vulnerable** | 33.63 (6.26) | 31.01 (5.69) | 28.50 (6.42) | 0.76 | <0.001 |
| ***Number(%)*** | 356 (10.5) | 379 (11.5) | 349 (10.4) |  |  |
| **MCS** |  |  |  |  |  |
| **Healthy** | 53.19 (5.32) | 54.69 (5.72) | 58.31 (5.19) | <0.001 | <0.001 |
| ***Number(%)*** | 2897 (85.4) | 2972 (86.7) | 2910 (86.4) |  |  |
| **Recovering** | 29.85 (5.73) | 46.83 (11.29) | 54.39 (6.93) | <0.001 | <0.001 |
| ***Number(%)*** | 282 (8.3) | 231 (6.7) | 234 (6.9) |  |  |
| **At risk** | 47.74 (11.56) | 40.56 (11.65) | 32.12 (5.97) | <0.001 | <0.001 |
| ***Number(%)*** | 212 (6.2) | 226 (6.6) | 224 (6.6) |  |  |
