# Supplementary material for: Trajectories and predictors of women’s health-related quality of life during pregnancy: A large longitudinal cohort study
Source: PLoS One. 2018 Apr 3;13(4):e0194999. doi: 10.1371/journal.pone.0194999 (PMC5882096; doi:10.1371/journal.pone.0194999)
Supplement: S3 Table — (DOCX) [file pone.0194999.s005.docx]

**S3 Table**

|  | **Healthy** | **Recovering** | **At risk** | **Vulnerable** | **P value** |
| --- | --- | --- | --- | --- | --- |
| **Maternal age at intake (years)** | 31.45±4.39 | 31.13±4.57 | 30.85±4.71 | 31.03±4.75 | 0.016 |
| **Gestational age at intake (weeks)** | 14.40±3.59 | 14.53±3.66 | 14.34±3.52 | 15.22±4.60 | <0.001 |
| **Maternal educational level** |  |  |  |  | <0.001 |
| **High** | 879 (35.7) | 170 (33.7) | 135 (26.5) | 101 (24.3) |  |
| **Mid-high** | 602 (24.5) | 135 (26.8) | 135 (26.5) | 90 (21.6) |  |
| **Mid-low** | 576 (23.4) | 124 (24.6) | 161 (31.6) | 143 (34.4) |  |
| **Low** | 402 (16.3) | 75 (14.9) | 78 (15.3) | 82 (19.7) |  |
| **Marital status** |  |  |  |  | 0.09 |
| **married/cohabiting** | 2229 (92.2) | 467 (93.6) | 472 (94.6) | 374 (90.6) |  |
| **Single** | 189 (7.8) | 32 (6.4) | 27 (5.4) | 39 (9.4) |  |
| **Parity** |  |  |  |  | <0.001 |
| **nullpara** | 1503 (60.5) | 332 (65.9) | 285 (55.3) | 224 (53.0) |  |
| **multipara** | 980 (39.50) | 172 (34.1) | 230 (44.7) | 199 (47.0) |  |
| **Monthly household income (€)** |  |  |  |  | <0.001 |
| **≤2200** | 521 (23.6) | 113 (25.5) | 149 (31.4) | 144 (39.7) |  |
| **>2200** | 1688 (76.4) | 330 (74.5) | 326 (68.6) | 219 (60.3) |  |
| **Planned pregnancy** |  |  |  |  | 0.27 |
| **No** | 425 (18.3) | 83 (16.8) | 99 (20.6) | 83 (21.0) |  |
| **Yes** | 1898 (81.7) | 410 (83.2) | 382 (79.4) | 312 (79.0) |  |
| **BMI at intake** | 23.98±3.81 | 24.27±4.25 | 25.16±4.38 | 25.49±5.11 | <0.001 |
| **Maternal smoking in early pregnancy** |  |  |  |  | 0.003 |
| **Non-smoker** | 1690 (74.1) | 395 (81.3) | 324 (69.8) | 290 (74.9) |  |
| **Smoked until pregnancy confirmed** | 286 (12.5) | 50 (10.3) | 73 (15.7) | 43 (11.1) |  |
| **Continued smoking in pregnancy** | 305 (13.4) | 41 (8.4) | 67 (14.4) | 54 (14.0) |  |
| **Maternal drinking during pregnancy** |  |  |  |  | <0.001 |
| **Teetotal** | 870 (38.0) | 227 (46.7) | 207 (44.4) | 192 (49.5) |  |
| **Drank until pregnancy confirmed** | 814 (35.6) | 169 (34.8) | 151 (32.4) | 128 (33.0) |  |
| **Continued drinking in pregnancy** | 604 (26.4) | 90 (18.5) | 108 (23.2) | 68 (17.5) |  |
| **Chronic conditions in previous year** |  |  |  |  | <0.001 |
| **None** | 1336 (60.4) | 250 (53.1) | 212 (47.3) | 152 (41.2) |  |
| **One** | 660 (29.8) | 151 (32.1) | 156 (34.8) | 135 (36.6) |  |
| **≥ two** | 217 (9.8) | 70 (14.9) | 80 (17.9) | 82 (22.2) |  |
| **Headache** |  |  |  |  | <0.001 |
| **Daily/ a few days a week** | 191 (8.5) | 76 (15.8) | 63 (13.8) | 98 (25.7) |  |
| **≤ once a week** | 2059 (91.5) | 405 (84.2) | 393 (86.2) | 283 (74.3) |  |
| **Sleeping badly** |  |  |  |  | <0.001 |
| **Daily** | 108 (4.8) | 42 (8.8) | 35 (7.6) | 69 (17.9) |  |
| **A few days a week** | 478 (21.3) | 136 (28.6) | 126 (27.3) | 121 (31.4) |  |
| **≤ once a week** | 1663 (73.9) | 298 (62.6) | 301 (65.2) | 195 (50.6) |  |
| **Fatigue** |  |  |  |  | <0.001 |
| **Daily** | 743 (32.7) | 279 (57.6) | 236 (51.1) | 259 (66.9) |  |
| **A few days a week** | 1039 (45.8) | 175 (36.2) | 179 (38.7) | 104 (26.9) |  |
| **≤ once a week** | 487 (21.5) | 30 (6.2) | 487 (21.5) | 47 (10.2) |  |
| **Pelvic pain** |  |  |  |  | <0.001 |
| **Daily/ a few days a week** | 57 (2.5) | 30 (6.2) | 47 (10.2) | 75 (19.9) |  |
| **≤ once a week** | 2211 (97.5) | 451 (93.8) | 418 (89.8) | 302 (80.1) |  |
| **Back pain** |  |  |  |  | <0.001 |
| **Daily** | 70 (3.1) | 43 (8.9) | 27 (5.8) | 81 (21.1) |  |
| **A few days a week** | 245 (10.8) | 88 (18.2) | 105 (22.7) | 87 (22.7) |  |
| **≤ once a week** | 1956 (86.1) | 352 (72.9) | 330 (71.4) | 215 (56.1) |  |
| **Nausea** |  |  |  |  |  |
| **Daily** | 476 (21.0) | 215 (44.3) | 142 (30.7) | 175 (45.1) |  |
| **A few days a week** | 645 (28.4) | 146 (30.1) | 134 (29.0) | 97 (25.0) |  |
| **≤ once a week** | 1150 (50.6) | 124 (25.6) | 186 (40.3) | 116 (29.9) |  |
| **Vomiting** |  |  |  |  | <0.001 |
| **Daily** | 75 (3.3) | 40 (8.2) | 21 (4.6) | 42 (11.0) |  |
| **A few days a week** | 173 (7.6) | 70 (14.4) | 40 (8.8) | 52 (13.6) |  |
| **≤ once a week** | 2019 (89.1) | 375 (77.4) | 394 (86.6) | 288 (75.4) |  |
| **Pregnancy-specific anxiety** | 0.73±0.32 | 0.81±0.32 | 0.78±0.31 | 0.86±0.33 | <0.001 |
