# Supplementary material for: Trajectories and predictors of women’s health-related quality of life during pregnancy: A large longitudinal cohort study
Source: PLoS One. 2018 Apr 3;13(4):e0194999. doi: 10.1371/journal.pone.0194999 (PMC5882096; doi:10.1371/journal.pone.0194999)
Supplement: S4 Table — (DOCX) [file pone.0194999.s006.docx]

**S4 Table**

|  | **Healthy** | **Recovering** | **At risk** | **P value** |
| --- | --- | --- | --- | --- |
| **Maternal age at intake (years)** | 31.35±4.41 | 30.85±4.66 | 30.85±5.35 | 0.05 |
| **Gestational age at intake (weeks)** | 14.50±3.74 | 14.38±3.72 | 14.71±3.64 | 0.56 |
| **Maternal educational level** |  |  |  | <0.001 |
| **High** | 1161 (34.7) | 79 (26.8) | 45 (18.1) |  |
| **Mid-high** | 818 (24.5) | 82 (27.8) | 62 (25.0) |  |
| **Mid-low** | 858 (25.7) | 78 (26.4) | 68 (27.4) |  |
| **Low** | 508 (15.2) | 56 (19.0) | 73 (29.4) |  |
| **Marital status** |  |  |  | <0.001 |
| **Married/cohabiting** | 3093 (94.0) | 256 (87.4) | 193 (79.1) |  |
| **Single** | 199 (6.0) | 37 (12.6) | 51 (20.9) |  |
| **Parity** |  |  |  | 0.12 |
| **Nullpara** | 2001 (59.2) | 193 (65.4) | 150 (59.5) |  |
| **Multipara** | 1377 (40.8) | 102 (34.6) | 102 (40.5) |  |
| **Monthly household income (€)** |  |  |  | <0.001 |
| **≤2200** | 724 (24.0) | 87 (35.1) | 116 (51.1) |  |
| **>2200** | 2291 (76.0) | 161 (64.9) | 111 (48.9) |  |
| **Planned pregnancy** |  |  |  | <0.001 |
| **No** | 516 (16.3) | 80 (27.8) | 94 (39.7) |  |
| **Yes** | 2651 (83.7) | 208 (72.2) | 143 (60.3) |  |
| **BMI at intake** | 24.29±4.09 | 24.49±4.44 | 24.75±4.40 | 0.20 |
| **Maternal smoking in early pregnancy** | | | | <0.001 |
| **Non-smoker** | 2392 (77.2) | 175 (60.6) | 132 (57.6) |  |
| **Smoked until pregnancy confirmed** | 360 (11.6) | 61 (21.1) | 31 (13.5) |  |
| **Continued smoking in pregnancy** | 348 (11.2) | 53 (18.3) | 66 (28.8) |  |
| **Maternal drinking in early pregnancy** | | | | 0.40 |
| **Teetotal** | 1290 (41.5) | 105 (36.2) | 101 (43.7) |  |
| **Drank until pregnancy confirmed** | 1073 (34.5) | 113 (39.0) | 76 (32.9) |  |
| **Continued drinking in pregnancy** | 744 (23.9) | 72 (24.8) | 54 (23.4) |  |
| **Chronic conditions** |  |  |  | 0.003 |
| **None** | 1706 (56.8) | 137 (49.8) | 107 (47.8) |  |
| **One** | 927 (30.9) | 101 (36.7) | 74 (33.0) |  |
| **≥ Two** | 369 (12.3) | 37 (13.5) | 43 (19.2) |  |
| **Headache** |  |  |  | <0.001 |
| **Daily/a few days a week** | 319 (10.4) | 57 (20.2) | 52 (22.6) |  |
| **≤ Once a week** | 2737 (89.6) | 225 (79.8) | 178 (77.4) |  |
| **Fatigue** |  |  |  | <0.001 |
| **Daily** | 1219 (39.5) | 162 (56.6) | 136 (59.4) |  |
| **Few days a week** | 1334 (43.2) | 94 (32.9) | 69 (30.1) |  |
| **≤ once a week** | 534 (17.3) | 30 (10.5) | 24 (10.5) |  |
| **sleeping badly** |  |  |  | <0.001 |
| **Daily** | 171 (5.6) | 44 (7.1) | 39 (17.1) |  |
| **Few days a week** | 695 (22.7) | 87 (30.4) | 79 (34.6) |  |
| **≤ Once a week** | 2192 (71.7) | 155 (54.2) | 110 (48.2) |  |
| **Pelvic pain** |  |  |  | 0.024 |
| **Daily/a few days a week** | 167 (5.4) | 20 (7.0) | 22 (9.6) |  |
| **≤ Once a week** | 2905 (94.6) | 266 (93.0) | 208 (90.4) |  |
| **Back pain** |  |  |  | <0.001 |
| **Daily** | 160 (5.2) | 25 (8.7) | 36 (15.7) |  |
| **Few days a week** | 431 (14.0) | 49 (17.0) | 45 (19.7) |  |
| **≤ Once a week** | 2491 (80.8) | 214 (74.3) | 148 (64.6) |  |
| **Nausea** |  |  |  | <0.001 |
| **Daily** | 806 (26.1) | 117 (40.8) | 85 (37.0) |  |
| **Few days a week** | 880 (28.5) | 80 (27.9) | 65 (27.0) |  |
| **≤ Once a week** | 1403 (45.4) | 90 (31.4) | 83 (36.1) |  |
| **Vomiting** |  |  |  | <0.001 |
| **Daily** | 132 (4.3) | 23 (8.0) | 23 (10.0) |  |
| **Few days a week** | 267 (8.7) | 40 (14.0) | 28 (12.2) |  |
| **≤ Once a week** | 2675 (87.0) | 223 (78.0) | 178 (77.7) |  |
| **Pregnancy-specific anxiety** | 0.73 ±0.30 | 0.93 ±0.38 | 0.96 ±0.35 | <0.001 |
