# Supplementary material for: Trajectories and predictors of women’s health-related quality of life during pregnancy: A large longitudinal cohort study
Source: PLoS One. 2018 Apr 3;13(4):e0194999. doi: 10.1371/journal.pone.0194999 (PMC5882096; doi:10.1371/journal.pone.0194999)
Supplement: S5 Table — (DOCX) [file pone.0194999.s007.docx]

S5 Table

|  | **Women included in analyses (n=2852)** | **Women excluded from analyses (n=1084)** | **P value** |
| --- | --- | --- | --- |
|  |  |  |  |
| **Maternal age at intake** | 31.5 (4.3) | 30.8 (5.0) | <0.001 |
| **Gestational age at intake** | 14.5 (3.7) | 14.4 (3.8) | 0.45 |
| **Maternal educational level** |  |  |  |
| **High** | 1023 (35.9) | 262 (25.3) | <0.001 |
| **Mid-high** | 760 (26.6) | 202 (19.5) |  |
| **Mid-low** | 701 (24.6) | 302 (29.2) |  |
| **Low** | 368 (12.9) | 269 (26.0) |  |
| ***missing*** | *0* | *48* |  |
| **Parity** |  |  |  |
| **Nullpara** | 1710 (60.0) | 634(59.1) | 0.62 |
| **Multipara** | 1142 (40.0) | 439 (40.9) |  |
| ***missing*** | *0* | *11* |  |
| **Monthly household income (€)** |  |  |  |
| **≤2200** | 723 (25.4) | 204 (32.0) | 0.006 |
| **>2200** | 2129 (74.6) | 434 (68.0) |  |
| ***missing*** | *0* | *446* |  |
| **BMI at intake** | 24.21 (3.95) | 24.68 (4.60) | 0.002 |
| ***missing*** | *0* | *18* |  |
| **Maternal smoking in early pregnancy** |  |  |  |
| **Non-smoker** | 2205 (77.3) | 494 (64.5) | <0.001 |
| **Smoked until pregnancy confirmed** | 336 (11.8) | 116 (15.1) |  |
| **Continued smoking in pregnancy** | 311 (10.9) | 156 (20.4) |  |
| ***missing*** | *0* | *318* |  |
| **Maternal drinking in early pregnancy** |  |  |  |
| **Teetotal during pregnancy** | 1110 (38.9) | 386 (49.7) | <0.001 |
| **Drank until pregnancy confirmed** | 1008 (35.6) | 254 (32.7) |  |
| **Continued drinking in pregnancy** | 734 (25.7) | 136 (17.5) |  |
| ***Missing*** | *0* | *308* |  |
| **Chronic conditions in the previous year** |  |  |  |
| **None** | 1593 (55.9) | 357 (55.0) | <0.001 |
| **One** | 922 (32.3) | 180 (27.7) |  |
| **≥ Two** | 337 (11.8) | 112 (17.3) |  |
| ***missing*** | *0* | *435* |  |
| **Headache** |  |  |  |
| **Daily/ Few days a week** | 310 (10.9) | 118 (16.5) | <0.001 |
| **≤ Once a week** | 2542 (89.1) | 598 (83.5) |  |
| ***missing*** | *0* | *368* |  |
| **Fatigue** |  |  |  |
| **Daily** | 1172 (41.1) | 345(46.0) | 0.02 |
| **Few days a week** | 1194 (41.9) | 303 (40.4) |  |
| **≤ Once a week** | 486 (17.0) | 102 (13.6) |  |
| ***missing*** | *0* | *334* |  |
| **Sleeping badly** |  |  |  |
| **Daily** | 171 (6.0) | 83 (11.5) | <0.001 |
| **Few days a week** | 670 (23.5) | 191 (26.5) |  |
| **≤ Once a week** | 2011 (70.5) | 446 (61.9) |  |
| ***missing*** | *0* | *364* |  |
| **Pelvic pain** |  |  |  |
| **Daily/ Few days a week** | 160 (5.6) | 49 (6.7) | 0.28 |
| **≤ Once a week** | 2692 (94.4) | 687 (93.3) |  |
| ***missing*** | *0* | *348* |  |
| **Back pain** |  |  |  |
| **Daily** | 156 (5.5) | 65 (8.7) | <0.001 |
| **Few days a week** | 378 (13.3) | 147 (19.7) |  |
| **≤ Once a week** | 2318 (81.3) | 535 (71.6) |  |
| ***missing*** | *0* | *337* |  |
| **Nausea** |  |  |  |
| **Daily** | 760 (26.6) | 248 (32.9) | <0.001 |
| **Few days a week** | 828 (29.0) | 194 (25.7) |  |
| **≤ Once a week** | 1264 (44.3) | 312 (41.4) |  |
| ***missing*** | *0* | *330* |  |
| **Vomiting** |  |  |  |
| **Daily** | 115 (4.0) | 63 (8.5) | <0.001 |
| **Few days a week** | 259 (9.1) | 76 (10.3) |  |
| **≤ Once a week** | 2478 (86.9) | 598 (81.1) |  |
| ***missing*** | *0* | *347* |  |
| **Pregnancy-specific anxiety** | 0.75 (0.31) | 0.81 (0.35) | <0.001 |
| ***missing*** | *0* | *371* |  |
