# Supplementary material for: Trajectories and predictors of women’s health-related quality of life during pregnancy: A large longitudinal cohort study
Source: PLoS One. 2018 Apr 3;13(4):e0194999. doi: 10.1371/journal.pone.0194999 (PMC5882096; doi:10.1371/journal.pone.0194999)
Supplement: S6 Table — (DOCX) [file pone.0194999.s008.docx]

S6 Table

|  | **Women included in analyses (n=2803)** | **Women excluded from analyses (n=1133)** | **P value** |
| --- | --- | --- | --- |
|  |  |  |  |
| **Maternal age at intake** | 31.5 (4.3) | 30.9 (4.9) | <0.001 |
| **Maternal educational level** |  |  |  |
| **High** | 1010 (36.0) | 276 (25.4) | <0.001 |
| **Mid-high** | 741 (26.4) | 313 (28.8) |  |
| **Mid-low** | 691 (24.7) | 221 (20.4) |  |
| **Low** | 361 (12.9) | 275 (25.3) |  |
| ***missing*** | *0* | *48* |  |
| **Marital status** |  |  |  |
| **Married/cohabiting** | 2625 (93.6) | 917 (89.4) | <0.001 |
| **Single** | 178 (6.4) | 109 (10.6) |  |
| ***missing*** | *0* | *107* |  |
| **Monthly household income (€)** |  |  |  |
| **≤2200** | 711(25.4) | 216 (31.4) | 0.001 |
| **>2200** | 2092 (74.6) | 471 (68.6) |  |
| ***missing*** | *0* | *446* |  |
| **Planned pregnancy** |  |  |  |
| **No** | 474 (16.9) | 216 (24.3) | 0.002 |
| **Yes** | 2329 (83.1) | 673 (75.7) |  |
| ***missing*** | *0* | *244* |  |
| **Maternal smoking in early pregnancy** |  |  |  |
| **Non-smoker** | 2435 (86.9) | 533 (65.4) | <0.001 |
| **Smoked until pregnancy confirmed** | 332 (11.8) | 120 (14.7) |  |
| **Continued smoking in pregnancy** | 305 (10.9) | 162 (19.9) |  |
| ***missing*** | *0* | *318* |  |
| **Chronic conditions in the previous year** |  |  |  |
| **None** | 1568 (55.9) | 382 (33.7) | 0.004 |
| **One** | 901 (32.1) | 201 (28.8) |  |
| **≥ Two** | 334 (11.9) | 115 (16.5) |  |
| ***missing*** | *0* | *435* |  |
| **Headache** |  |  |  |
| **Daily/ Few days a week** | 302 (10.8) | 126 (16.5) | <0.001 |
| **≤ Once a week** | 2501 (89.2) | 639 (83.5) |  |
| ***missing*** | *0* | *368* |  |
| **Fatigue** |  |  |  |
| **Daily** | 1152 (41.1) | 365 (45.7) | 0.04 |
| **Few days a week** | 1176 (42.0) | 321 (40.2) |  |
| **≤ Once a week** | 475 (16.9) | 113 (14.1) |  |
| ***missing*** | *0* | *334* |  |
| **Sleeping badly** |  |  |  |
| **Daily** | 167 (6.0) | 87 (11.3) | <0.001 |
| **Few days a week** | 659 (23.5) | 202 (26.3) |  |
| **≤ Once a week** | 1977 (70.5) | 480 (62.4) |  |
| ***missing*** | *0* | *364* |  |
| **Pelvic pain** |  |  |  |
| **Daily/ Few days a week** | 157 (5.6) | 52 (6.6) | 0.28 |
| **≤ Once a week** | 2646 (94.4) | 733 (93.4) |  |
| ***missing*** | *0* | *348* |  |
| **Back pain** |  |  |  |
| **Daily** | 155 (5.5) | 66 (8.3) | <0.001 |
| **Few days a week** | 374 (13.3) | 151 (19.0) |  |
| **≤ Once a week** | 2274 (81.1) | 579 (72.7) |  |
| ***missing*** | *0* | *337* |  |
| **Nausea** |  |  |  |
| **Daily** | 752 (26.8) | 256 (31.9) | 0.01 |
| **Few days a week** | 816 (29.1) | 206 (25.7) |  |
| **≤ Once a week** | 1235 (44.1) | 341 (42.5) |  |
| ***missing*** | *0* | *330* |  |
| **Vomiting** |  |  |  |
| **Daily** | 114 (4.1) | 64 (8.1) | <0.001 |
| **Few days a week** | 254 (9.1) | 81 (10.3) |  |
| **≤ Once a week** | 2435 (86.8) | 641 (81.6) |  |
| ***missing*** | *0* | 347 |  |
| **Pregnancy-specific anxiety** | 0.75 (0.31) | 0.80 (0.34) | <0.001 |
| ***missing*** | *0* | *371* |  |
