# Supplementary material for: Trajectories and predictors of women’s health-related quality of life during pregnancy: A large longitudinal cohort study
Source: PLoS One. 2018 Apr 3;13(4):e0194999. doi: 10.1371/journal.pone.0194999 (PMC5882096; doi:10.1371/journal.pone.0194999)
Supplement: S7 Table — (DOCX) [file pone.0194999.s009.docx]

S7 Table

| **Physical HRQOL trajectories** | **Women included in analyses (n=2852)** | **Women excluded from analyses (n=1084)** | **P value** |
| --- | --- | --- | --- |
| **Healthy** | 1813 (63.6) | 678 (62.5) | 0.18 |
| **Recovering** | 375 (13.1) | 130 (12.0) |  |
| **At risk** | 375 (13.1) | 141 (13.0) |  |
| **Vulnerable** | 289 (10.1) | 135 (12.5) |  |
