# Supplementary material for: Trajectories and predictors of women’s health-related quality of life during pregnancy: A large longitudinal cohort study
Source: PLoS One. 2018 Apr 3;13(4):e0194999. doi: 10.1371/journal.pone.0194999 (PMC5882096; doi:10.1371/journal.pone.0194999)
Supplement: S8 Table — (DOCX) [file pone.0194999.s010.docx]

S8 Table

| **Mental HRQOL trajectories** | **Women included in analyses (n=2803)** | **Women excluded in analyses (n=1133)** | **P value** |
| --- | --- | --- | --- |
| **Healthy** | 2406 (85.8) | 982 (86.7) | 0.80 |
| **Recovering** | 214 (7.6) | 81 (7.1) |  |
| **At risk** | 183 (6.5) | 70 (6.2) |  |
